# Early career choices and successful career progression in surgery in the UK: prospective cohort studies

**DOI:** 10.1186/1471-2482-10-32

**Published:** 2010-11-02

**Authors:** Michael J Goldacre, Louise Laxton, Ewen M Harrison, Jennifer MJ Richards, Trevor W Lambert, Rowan W Parks

**Affiliations:** 1 UK Medical Careers Research Group, Department of Public Health, Oxford University, Oxford, UK; 2 Clinical and Surgical Sciences (Surgery), University of Edinburgh, Edinburgh, UK

## Abstract

**Background:**

Changes to the structure of medical training worldwide require doctors to decide on their career specialty at an increasingly early stage after graduation. We studied trends in career choices for surgery, and the eventual career destinations, of UK graduates who declared an early preference for surgery.

**Methods:**

Postal questionnaires were sent, at regular time intervals after qualification, to all medical qualifiers from all UK medical schools in selected qualification years between 1974 and 2005. They were sent in the first year after qualification, at year three and five years after qualification, and at longer time intervals thereafter.

**Results:**

Responses were received from 27 749 of 38 280 doctors (73%) at year one, 23 468 of 33151 (71%) at year three, and 17 689 of 24 870 (71%) at year five. Early career preferences showed that surgery has become more popular over the past two decades. Looking forward from early career choice, 60% of respondents (64% of men, 48% of women) with a first preference for a surgical specialty at year one eventually worked in surgery (p < 0.001 for the male-female comparison). Looking backward from eventual career destinations, 90% of responders working in surgery had originally specified a first choice for a surgical specialty at year one. 'Match' rates between eventual destinations and early choices were much higher for surgery than for other specialties. Considering factors that influenced early specialty choice 'a great deal', comparing aspiring surgeons and aspiring general practitioners (GPs), a significantly higher percentage who chose surgery than general practice specified enthusiasm for the specialty (73% vs. 53%), a particular teacher or department (34% vs. 12%), inclinations before medical school (20% vs. 11%), and future financial prospects (24% vs. 13%); and a lower percentage specified that hours and working conditions had influenced their choice (21% vs. 71%). Women choosing surgery were influenced less than men by their inclinations before medical school or by their future financial prospects.

**Conclusions:**

Surgery is a popular specialty choice in the UK. The great majority of doctors who progressed in a surgical career made an early and definitive decision to do so.

## Background

At a time of change to postgraduate education programmes, as is currently taking place in the United Kingdom [1], North America [2,3], and Australasia [4], it is important to know about the timing of junior doctors' definitive career choices. In addition to educational reform, there are trends to reduce working hours, to shorten the duration of specialist training and to streamline career progression. These changes require junior doctors to choose their career specialty at an increasingly early stage. Decisions about when specialist training in surgery should start, and the planning of postgraduate medical education and of the medical workforce more generally, must be informed by knowledge about junior doctors' early choices of career specialty, their level of certainty of choice, their career progression, and their eventual destinations.

For many years the UK Medical Careers Research Group has followed graduates from all medical schools in the United Kingdom [5-7]. Among other topics, doctors are asked about their choice of future career. Findings on the career choices of doctors in their early years after qualification have been published as they have emerged from each survey and have been communicated to policy makers in medical education and workforce planning. Survey findings have previously been summarised and published for a range of individual specialties [8-12].

The aim of this study was to describe the career intentions and eventual destinations of doctors in the surgical specialities. We report on trends over time in career choice and on factors that the doctors specified had influenced them in their choice of surgery. It is important to monitor career intentions in order to know what the aspirations of newly qualified doctors are; whether aspirations are changing; to identify variation in choice between different groups of doctors, such as the difference between men and women, and whether differences have changed over time; and to determine whether the percentage of newly qualified doctors who aspire to practise in each specialty is insufficient, sufficient, or in excess of likely service requirements for the specialty. We were particularly interested in the stage of training at which doctors made a definitive decision to pursue a career in surgery and how this related to the timing of selection into surgical training schemes.

## Methods

The analysis in this paper is based on surveys of the UK graduation cohorts of 1974, 1977, 1980, 1983, 1993, 1996, 1999, 2000, 2002, and 2005. The methods used have been described in detail elsewhere [5,6]. Towards the end of the first, third and fifth years after graduation, and at longer time intervals after that, questionnaires were sent to all medical graduates in each year-of-graduation cohort. Up to four reminders were sent to non-respondents. The graduates of 1974 from all medical schools in England, Wales and Scotland were surveyed. From the cohorts of 1977 onwards, the surveys covered the whole of the UK including Northern Ireland. The total number of medical schools in the UK has changed across the decades covered by the surveys (notably, some of the London schools merged) but, at the time of analysis for this paper, there were 23 medical schools in the studies. The UK MCRG studies and surveys have ethical approval from the National Research Ethics Service (reference 04/Q1907/48) following approval by the Brighton, Mid Sussex and East Sussex local research ethics committee.

The doctors mailed in the first survey of each cohort comprised the whole cohort as it was at the time of qualification. Subsequent surveys of a cohort included all its original members except those doctors who elected not to participate, or were untraceable, or were known to have died. Initial addresses were obtained from the doctors' registration with the General Medical Council (GMC). For follow-up surveys, addresses supplied by doctors at each previous survey and/or those updated by the GMC or identified from the Medical Register or from the Medical Directory were used.

In the year one, three and five surveys, each doctor was asked *"Have you made up your mind about your choice of long-term career?*", with the option to choose a response from '*definitely*', '*probably*' or '*not really*'. They were asked to indicate their specialty choice in their own words and to be 'as general or as specific' as they wished. Many doctors who wanted to become surgeons specified in their responses simply 'surgery' or 'general surgery' in the first postgraduate year and then, increasingly, cited individual specialties or subspecialties within surgery in later years. In all surveys, doctors were asked to provide details of their current post and previous posts since they last responded.

For the purpose of this paper, surgical specialties were grouped together. The following were included in our analysis of surgical specialties: surgery (without further specification); general surgery (which included general surgery, specified as such, either 'surgery' or 'general surgery' combined with a specified interest in a sub-specialty, and respondents specifying academic surgery); otorhinolaryngology; neurosurgery; ophthalmology; trauma and orthopaedic surgery; paediatric surgery; plastic surgery; cardiothoracic surgery; vascular surgery; oral and maxillofacial surgery; urology; and 'other surgery' (consisting of data recorded before 1993 as 'surgical sub-specialties' without further detail; and data recorded after 1993 as colorectal surgery, gastrointestinal surgery, head & neck surgery, hand surgery and renal transplant surgery). The surgical specialties, thus defined, were compared, as a group, with all other respondents, or, when relevant, with choices for other hospital-based specialties combined and for general practice.

If doctors had more than one choice of specialty, they were asked to list up to three in order of preference and to indicate whether their choices were of equal preference (in which case they are termed 'tied choices').

The main focus of our analysis in this paper was on doctors who specified that surgery was their first choice of career (including tied choices unless otherwise specified); but we also show some data on doctors who specified surgery as a second or third choice.

Data were analysed by bivariate cross-tabulation with chi-squared tests and adjusted residuals. Binary logistic regression was used for multi-factorial adjustments. When multiple comparisons of data were made, a *P*-level of <0.01 was regarded as evidence of a statistically significant result.

## Results

### Response Rates

In the first year after qualification, the survey questionnaires were sent to a total of 38 280 UK doctors covering all ten cohorts. A total of 27 749 doctors (72.5%) replied. Three years after qualification, the survey questionnaire was sent to 33 151 UK doctors covering the first nine cohorts (1974-2002) and 23 468 (70.8%) replied. Five years after qualification, covering the seven cohort surveys from 1974-2000, 17 689 from a possible 24 870 doctors (71.1%) replied.

### Choice of long-term career

**Figure 1:**
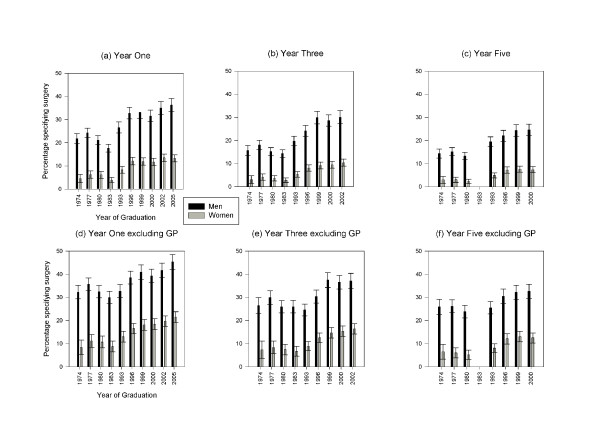
UK Doctors who specified surgical specialties as their first choice of eventual career*. Footnotes: Percentages (with 95% confidence intervals) in each year after graduation, first choices for surgery including tied first choices, showing (a to c) percentages of all respondents, and (d to f) percentages of all respondents excluding those who specified general practice as their first choice. Figures 1a-1c: Numbers of respondents: 14 345 (men), 13 404 (women), 27 749 (total) in Year One; 12 420 (men), 11 048 (women), 23 468 (total) in Year Three; 9 561(men), 8 128 (women), 17 689 (total) in Year Five. Chi square tests (χ^2^_1_) comparing first choice for surgical specialties made by men and women (all graduation years combined): Year One 1293.7; Year Three 919.7; Year Five 658.5; all years *P <*0.001. Figures 1d-1f: Numbers of respondents excluding those with first choices for GP: 10 567 (men), 8 331 (women), 18 898 (total) in Year One; 8 569 (men), 6 441 (women), 15 010 (total) in Year Three; 6 307 (men), 4 557 (women), 10 864 (total) in Year Five. Chi square tests (χ^2^_1_) comparing first choice for surgical specialties made by men and women (all graduation years combined) excluding GP: Year One 968.5; Year Three 706.8; Year Five 523.6; all years *P <*0.001.

Figure 1a-c shows trends in the popularity of the surgical specialties as a first choice of long-term career. Combining the cohorts of 1974, 1977 and 1980, 22.4% of men and 5.8% of women selected surgery as their first choice of career at the end of the first year after graduation. This fell to 17.7% of men and 3.9% of women in the cohort of 1983. At this time, general practice had become a very popular career choice (between the 1970s and 1983 choices for the hospital medical specialties, as well as the surgical specialties, declined). Between 1983 and 1993, the popularity of general practice fell and that of surgery increased substantially. Choices for surgery remained high in the cohorts from 1993-2005: in these cohorts 32.5% of men and 12.0% of women specified in year one that surgery was their first choice of eventual career. The choices of the latest cohort studied in year one (2005) showed little change in the percentages of men and women choosing surgery as their first choice compared with the 2002 cohort (2002 cohort: men 35.0%, women 13.5%, compared with, respectively, 36.3% and 13.3% in the 2005 cohort). Similarly, trends over time in the percentage of doctors choosing surgery at three and five years after qualification showed a gradual fall from 1974 to 1983, then a substantial rise to 1993, and a consistent level since then (Figure 1a-c).

The percentage of doctors who specified that surgery was their first choice of career fell between year one and year three, and fell further between year three and year five (Figure 1a-c). In year one, for all cohorts combined, 27.0% of men and 10.1% of women gave surgical specialties as their long-term career choice. At the end of year three this fell to 21.0% of men and 7.0% of women, and at year five fell further to 18.5% and 5.6% respectively.

### Trends in choices for surgery among men and women

Although surgery remained a much more popular career choice for men than for women, the gap reduced across successive cohorts. Considering responses at year one in the 1974 cohort, the percentage of men who chose surgery was 4.8 times the percentage of women. This ratio reduced to 3.2 in the 1993 cohort and to 2.7 in the 2005 cohort. Comparing choices of men and women at the end of year five, the gap was larger. At year five, within the 1974 cohort the percentage of men who chose surgery outnumbered that of women by 4.8 to 1. By 1993 this reduced to 4.0 to 1 and by 2000 (the latest cohort surveyed by us at five years) it had reduced further to 3.3 to 1.

### Percentages of doctors who chose the surgical specialties, excluding doctors who chose general practice

It is possible that, for some doctors, their primary decision is whether to seek a career in general practice and then to decide which hospital specialty they want to pursue. Choices for surgery were therefore re-calculated, omitting from the denominator those doctors whose first choice was for general practice (Figure 1d-f). This shows that the rise in popularity of surgery was not simply due to the general increase in the popularity of hospital practice; the percentage of doctors who chose surgery, expressed as a percentage of all who chose hospital practice, also increased.

### Certainty of career choice

The level of certainty expressed by doctors about their chosen specialty increased considerably between the first, third and fifth years after qualification (Table 1). At each successive survey of each cohort, a significantly higher percentage of doctors who expressed a first choice for surgery were certain of their choice in comparison with those who chose other hospital specialties; but in years one and three aspiring general practitioners were more certain than aspiring surgeons about their career choice (Table 1). Men who chose the surgical specialties were a little more certain than women about their career choice (Table 1).

**Table 1:** Firmness of choice of doctors who expressed a first preference for each specialty group*

		Year after qualification and career choice								
		
		Year One			Year Three			Year Five		
		
Group and response		Surgery	Other Hospital	General Practice	Surgery	Other Hospital	General Practice	Surgery	Other Hospital	General Practice
Percentages										
Percentage who had made up their mind:										
Men	Definitely	37	19	43	48	41	66	75	64	78
	Probably	47	54	45	44	46	30	22	31	19
	Not Really	16	27	12	8	14	4	3	5	2
	Total	100	100	100	100	100	100	100	100	100
	*P*-value^1^	-	<0.001	<0.001	-	<0.001	<0.001	-	<0.001	0.012
Women	Definitely	30	14	40	47	34	56	69	59	68
	Probably	48	51	48	42	47	39	26	34	28
	Not Really	22	34	12	11	19	5	5	7	4
	Total	100	100	100	100	100	100	100	100	100
	*P*-value^1^	-	<0.001	<0.001	-	<0.001	<0.001	-	<0.001	>0.05
Total	Definitely	35	17	41	48	38	61	73	61	73
	Probably	47	53	47	44	46	35	23	32	24
	Not Really	18	31	12	9	17	5	4	7	3
	Total	100	100	100	100	100	100	100	100	100
	*P*-value^1^	-	<0.001	<0.001	-	<0.001	<0.001	-	<0.001	>0.05
Denominators on which the percentages are based										
Men		3831	6491	3319	2593	5473	3682	1753	4123	3142
Women		1344	7206	4251	771	5360	4298	457	3739	3400
Total		5175	13697	7590	3364	10833	7980	2210	7682	6542

* Including tied first choices. Question asked: *"Have you made up your mind about your choice of long-term career?"*at year 1 to the 1974-2005 cohorts; at year 3 to the 1974-2002 cohorts; and to the 1974-1980 and 1993-2000 cohorts at year 5.

^1^*P*-values, based on chi-squared tests for the column in which the p-value appears, comparing certainty of choice (definitely, probably, not really) of those choosing the surgical specialties with those choosing the other hospital specialties, and with those choosing general practice.

Chi squared (χ^2^_2_) tests were also conducted to compare the certainty of choice of men choosing surgery with that of women choosing surgery: men were more certain than women in year one 31.9, *P <*0.001, year three 8.4, *P = *0.015, year five 8.6, *P = *0.013.

### Choice by medical school (Table 2)

**Table 2:** Percentages and numbers of graduates choosing surgery by clinical medical school

	Percentages (and numbers) expressing a preference for surgery in years 1, 3 and 5 after qualification					
	
Clinical Medical School	Year One		Year Three		Year Five	
	%	n	%	n	%	n
Birmingham	19.3	151	15.4	93	10.9	51
Bristol	22.6^H^	139	17.2	85	14.8	55
Cambridge	25.2	120	20.6	81	18.6	58
Leeds	19.8	141	17.7	101	13.8	65
Leicester	17.7	115	13.0	61	10.8	39
Liverpool	20.5	129	15.8	82	11.7	46
Manchester	23.7^H^	274	19.1	169	16.6	117
Newcastle	17.1	117	13.1	73	10.6	44
Nottingham	16.0^L^	102	13.9	78	13.2	55
Oxford	26.1^H^	123	22.4^H^	85	20.3^H^	57
Sheffield	19.4	146	13.4	77	11.5	47
Southampton	18.3	108	13.5	63	11.6	42
Imperial College	24.6^H^	258	18.9^H^	162	15.4	108
King's College	23.8^H^	285	19.9^H^	194	17.2^H^	132
Queen Mary & Westfield	23.3	178	18.3	113	16.8	89
St George's	23.7	144	17.3	78	14.8	54
University College London	20.5	235	16.4	154	13.7	102
Aberdeen	19.8	115	15.3	73	14.7	51
Dundee	19.3	98	16.6	66	12.8	38
Edinburgh	16.5^L^	149	15.3	110	11.9	66
Glasgow	19.7	169	14.4	101	13.1	70
Wales	21.9	160	16.9	90	13.3	56
Belfast	18.1	116	12.7^L^	68	11.5	45
Total	20.8	2572	16.5	2257	14.0	1487

Cohorts from 1993 onwards.

Graduates from the University of London have been aggregated according to current London medical schools. The 2005 graduates from Leicester/Warwick are included within Warwick.

Where the level of choices for surgery differs significantly (*P <*0.05) from the overall average, it is marked as low (L) or high (H).

Numbers of respondents: 17 142 in Year One, 13 675 in Year Three and 10 615 in Year Five. The medical school was unknown for a small number of respondents, who have been excluded from the total.

Binary logistic regression model: after adjustment for sex and the year of graduation, the Wald statistic (22 degrees of freedom) for medical school differences gave values of Year One 65.4, Year Three 45.5, Year Five 37.6 (all *P <*0.0001).

For the cohorts qualifying from 1993 onwards, we compared the percentages of graduates from each medical school who specified surgery as their first choice of career (Table 2). There were significant differences between medical schools in their graduates' choice of surgery, after adjusting the comparisons for the doctors' year of qualification and sex (Table 2, footnote). Graduates from Oxford and Kings College London showed higher than average choices for surgery in each of years one, three and five after qualification. In addition, significantly higher rates than average of surgical choices were found for graduates of Bristol (year one), Manchester (year one), and Imperial College London (years one and three). Medical schools with lower choices than average for the surgical specialties were Nottingham (year one), Edinburgh (year one), and Belfast (year three).

### Factors that influenced choice of surgery 'a great deal' (Table 3)

**Table 3:** Percentages of doctors specifying that each factor had influenced their specialty choice 'a great deal'

	Percentages								
	
	Year One (1993-2002)			Year Three (1993, 1996, 2002)			Year Five (1993-2000)		
Factor	Surgery	Other Hospital	GP	Surgery	Other Hospital	GP	Surgery	Other Hospital	GP
Men									
Domestic circumstances	8.6^4^	10.7	35.4	11.74	14.1	44.6	19.7^3,4^	24.6	61.9
Hours/working conditions	20.6^3,4^	33.1	71.2	27.2^3,4^	36.3	77.6	28.7^3,4^	40.9	83.3
Future financial prospects	24.0^1,2^	13.4	19.1	25.01	11.4	28.5	22.2^1^	11.8	24.1
Career & promotion prospects	29.0^2^	27.8	21.9	28.5	27.8	30.3	30.9^2^	30.4	26.5
Self-appraisal of own skills/aptitudes	49.0^2^	50.7	43.3	50.5	51.9	49.6	57.8^2^	57.4	49.9
Advice from others	16.8	16.0	15.6	19.2^1,2^	15.5	15.0	18.5^2^	14.9	11.7
Experience of chosen subject as a student	47.1^2^	44.5	35.6	26.9^2^	23.3	21.1	25.4^1,2^	21.3	16.5
A particular teacher/department^#^	33.9^1,2^	27.7	12.4	32.9^2^	29.0	12.6	35.1^1,2^	26.5	5.0
Inclinations before medical school	19.7^1,2^	11.4	14.5	20.2^1,2^	9.7	15.4	16.7^1,2^	9.2	11.1
Experience of jobs so far	56.9^1,2^	48.6	43.7	70.1^1,2^	64.5	49.6	75.3^1,2^	68.2	50.5
Enthusiasm/Commitment	73.3^1,2^	61.3	54.6	71.0^1,2^	60.5	55.7	84.1^1,2^	74.0	58.0
Women									
Domestic circumstances	9.5^3,4^	16.0	38.5	11.9^3,4^	20.5	50.6	26.2^3,4^	43.9	68.0
Hours/working conditions	25.4^3,4^	37.1	74.7	30.0^3,4^	42.2	83.6	37.9^3,4^	52.1	89.5
Future financial prospects	10.7^1,4^	7.6	14.3	7.4^4^	6.8	20.1	4.9^4^	5.3	14.5
Career & promotion prospects	23.1^2^	22.8	14.3	19.2	21.6	19.5	18.2	21.1	17.3
Self-appraisal of own skills/aptitudes	51.4	49.1	47.6	43.3^3,4^	51.9	50.1	59.0	59.2	54.0
Advice from others	19.5^2^	17.1	16.5	17.9	17.1	17.4	17.5^1,2^	14.1	10.6
Experience of chosen subject as a student	52.4^2^	47.6	37.4	36.7^1,2^	28.9	20.6	33.3^1,2^	23.3	18.1
A particular teacher/department^#^	43.1^1,2^	31.1	13.8	46.4^1,2^	31.3	10.4	35.8^1,2^	25.5	5.7
Inclinations before medical school	14.9	14.0	14.8	14.7	12.5	14.5	11.1	9.3	13.9
Experience of jobs so far	64.6^1,2^	51.9	50.3	74.4^2^	71.6	56.2	73.6^2^	70.6	55.1
Enthusiasm/Commitment	78.1^1,2^	67.3	61.2	77.3^1,2^	68.5	56.9	86.8^1,2^	78.7	63.1

^1^Surgery significantly higher percentage (p < 0.01), compared with responses from those who chose other hospital specialties

^2^Surgery significantly higher percentage, compared with responses from those who chose general practice

^3^Surgery significantly lower percentage, compared with responses from those who chose other hospital specialties

^4^Surgery significantly lower percentage, compared with responses from those who chose general practice.

^#^This statement was not presented to graduates of 1996 in their third year after graduating. Excluding this statement, the numbers of respondents to each statement varied between 2748 and 2774(year one), 1156 and 1166 (year three),1373 and 1380 (year five) of those whose first choice was surgery, between 7084 and 7156 (year one), 4017 and 4048 (year three), 5114 and 5159 (year five) of those choosing other hospital specialties, and of those choosing general practice between 3493 and 3522 (year one), 2199 and 2221 (year three) and 3257 and 3278 (year five).

Numbers on which this table is based are given in additional file 1.

In the text that follows, we have put the precise wording of statements in our questionnaires in quotation marks. For doctors whose first choice of career was surgery in year one, the factor scored by the highest percentage of doctors as having 'a great deal' of influence was 'enthusiasm/commitment: what I really want to do'. The differences between men and women were statistically significant: women scored more highly (78%) than men (73%) on this item. The statement on enthusiasm and commitment was scored significantly more highly by doctors who wanted a career in surgery (e.g. 73% of men) than by doctors seeking a career in other hospital specialties (61% of men) or in general practice (55% of men), Table 3.

The factor scoring the second highest percentage for aspiring surgeons was 'experience of jobs so far', which was a stronger influence for those who chose surgery (57% for men in year one, Table 3) than for those who chose careers in other hospital specialties (49%) or in general practice (44%).

Other important influencing factors were 'self-appraisal of own skills/aptitudes', 'experience of the subject as a student' and the influence of 'a particular teacher/department'. For the latter, there was a striking contrast between doctors hoping to enter surgery and those who chose other careers. In year one, 'a particular teacher/department' had 'a great deal' of influence on the choice of specialty for 34% of men hoping to enter surgery, compared with 28% of men opting for other hospital careers and 12% opting for general practice. The corresponding figures for women were 43%, 31% and 14%, respectively. 'Inclinations before entering medical school' were a more important influence for men choosing surgery (cited by 20% of men in year one) than the other hospital specialties (11%) or general practice (15%). They were less of an influence for women choosing surgery (15%); and were a similar level of influence as that for women choosing other hospital specialties (14%) or general practice (15%). 'Future financial prospects' were a more important influence for men choosing surgery (cited by 24% of men in year one) than for men who chose other hospital specialties (13%) or general practice (19%). They were less of an influence for women choosing surgery (11%).

Consideration by the respondents of their 'domestic circumstances' was an important factor in year one for about a third of doctors who wanted to enter general practice, rising to about a half in year three and two-thirds in year five. It was a much less important consideration for doctors in their choice of a career in surgery (Table 3). Similarly, considerations of 'hours/working conditions' were important for a substantial majority of doctors who sought a career in general practice (71% of men in year 1 and 75% of women); these considerations were much less important for the majority who sought careers in surgery (21% of men and 25% of women).

### Looking forwards: eventual destinations of doctors who expressed an early preference for surgery (Table 4)

**Table 4:** Looking forwards: percentage of doctors, whose original choice was surgery, who eventually practised as surgeons

	Career destination in surgery								
	
	Men			Women			Total		
	
Original Choice	%	n	N	%	n	N	%	n	N
Year 1									
Surgery untied 1st	64.1	1091	1701	47.8	239	500	60.4	1330	2201
Surgery tied 1st	29.5	46	156	21.4	12	56	27.4	58	212
Surgery 2nd or 3rd	14.2	45	318	7.5	11	147	12.0	56	465
Choices other than surgery†	1.0	59	5273	0.7	40	5855	0.9	99	11128
Year 3									
Surgery untied 1st	80.6	1120	1389	67.4	260	386	77.7	1380	1775
Surgery tied 1st	44.4	16	36	23.5	4	17	37.7	20	53
Surgery 2nd or 3rd	8.6	12	140	10.6	5	47	9.1	17	187
Choices other than surgery†	0.8	45	5611	0.3	15	5923	0.5	60	11534
Year 5*									
Surgery untied 1st	91.4	1015	1111	81.8	256	313	89.3	1271	1424
Surgery tied 1st	26.3	5	19	62.5	5	8	37.0	10	27
Surgery 2nd or 3rd	6.1	3	49	15.8	3	19	8.8	16	68
Choices other than surgery†	0.5	22	4701	0.2	10	5187	0.3	32	9888

The table covers all doctors who specified surgery as their choice of career at years one, three and five: percentages (and numbers) whose eventual career was in surgery at year ten (cohorts of 1974, 1977, 1983*, 1993, 1996) or seven (1999, 2000 cohorts)

† Includes all respondents not making an explicit choice for surgery.

* The 1983 cohort was not surveyed five years after graduation.

Table 4 shows the extent to which doctors who expressed an early career preference for surgery eventually practised in surgery. The results show career destinations ten years after qualification for the cohorts of 1974-1996, and seven years after for the cohorts of 1999 and 2000. In order to compare choices and destinations, the surgical specialties were grouped as a whole. Thus, for example, a respondent who chose general surgery at year one and was working in neurosurgery at year ten was judged to have chosen and to be working in 'the surgical specialties'. Table 4 shows early career preferences subdivided into untied first choices for the surgical specialties, tied first choices, second and third choices, and choices other than surgery. A tied choice (see Method) means that the doctor chose surgery and a specialty from outside surgery and gave them equal preference as their first choice of career.

Sixty-four per cent of men and 48% of women who gave an untied first choice for a surgical specialty at year one eventually worked in surgery. This rose to 81% of men and 67% of women who gave an untied first choice for surgery at year three, and to 91% of men and 82% of women with untied first choices at year five, who worked eventually in surgery. At each year surveyed, comparing men and women with an untied first choice for surgery, men were significantly more likely than women who initially chose surgery to be working in surgery eventually (Table 4; chi squared tests for choices at year one χ^2^_1 _= 43.1, n = 2201; year three χ^2^_1 _= 30.8, n = 1775; year five χ^2^_1 _= 23.3, n = 1424, all *P <*0.001).

Respondents who gave tied first choices for surgery were much less likely to work eventually in surgery than those who gave a sole choice for surgery; and those who specified surgery as a second or third choice of career were even less likely to work in surgery eventually (Table 4).

We split the analysis into the more recent cohorts (1996, 1999, 2000) and the earlier cohorts (1974-1993). The predictive power of early choice, in 'matching' with eventual destinations in surgery, was similar in the two groups. For example, the percentage of aspiring surgeons in year 1 who eventually progressed into surgery was 62.0% (619/998) for doctors in the later cohorts and 59.1% (711/1203) in the earlier; it was 76.7% (650/847) for year 3 choices in the later cohorts and 78.7% (730/928) in the earlier; and it was 90.3% (674/746) for year 5 choices in the later cohorts and 88.1% (597/678) in the earlier cohorts.

### Looking backwards: practising surgeons and their earlier career choices (Table 5)

**Table 5:** Looking backwards: percentage of doctors, whose eventual career was surgery, who originally gave each choice

	Career destination in surgery					
	**Men**		**Women**		**Total**	
	
**Original Choice**	**%**	**n**	**%**	**n**	**%**	**n**

Year 1						
Surgery untied 1st	87.9	1091	79.1	239	86.2	1330
Surgery tied 1st	3.7	46	4.0	12	3.8	58
Surgery 2nd or 3rd	3.6	45	3.6	11	3.6	56
Choices other than surgery	4.8	59	13.2	40	6.4	99
Total	100.0	1241	100.0	302	100.0	1543
Year 3						
Surgery untied 1st	93.9	1120	91.5	260	93.4	1380
Surgery tied 1st	1.3	16	1.4	4	1.4	20
Surgery 2nd or 3rd	1.0	12	1.8	5	1.2	17
Choices other than surgery	3.8	45	5.3	15	4.1	60
Total	100.0	1193	100.0	284	100.0	1477
Year 5						
Surgery untied 1st	97.1	1015	93.4	256	96.4	1271
Surgery tied 1st	0.5	5	1.8	5	0.8	10
Surgery 2nd or 3rd	0.3	3	1.1	3	1.2	16
Choices other than surgery	2.1	22	3.6	10	2.4	32
Total	100.0	1045	100.0	274	100.0	1319

Table includes all respondents working in surgery at year ten (cohorts of 1974, 1977, 1983, 1993, 1996) or seven (cohorts of 1999, 2000): percentages and numbers of men and women whose original choice was for each specialty group at years one, three and five

Most practising surgeons had made an unambiguous decision to become surgeons by the first year after qualifying as doctors: 88% of men and 79% of women who eventually practised in surgery had specified a surgical career as their sole first choice of career in their first post-qualification year (Table 5). A further 4% of men and of women had given surgery as a tied first choice. Another 4% of men and of women had given surgery as a second or third choice. Only 5% of men, but 13% of women, who were surgeons had specified a non-surgical career choice in year one (Table 5; χ^2^1 = 27.8, comparing men and women, n = 1543, *P *< 0.001). Ninety-four per cent of men and 92% of women in surgery had specified surgery as their sole first choice of career when they were in year three; and 97% of men and 93% of women had specified surgery when they were in year five (Table 5).

## Discussion

The surveys analysed by us are unique: there are no other comparable studies of doctors' career intentions and progression on the scale of ours, over the period covered by us, in the UK. An important strength of our studies is that they are prospective: for example, the data on the early postgraduate career choices of current practising surgeons were specified contemporaneously. Our studies are therefore free from recall bias, including any post-hoc reconstruction by the doctors of their original intentions.

### Eventual destinations in surgery, early intentions, and their relevance to timing of selection into surgery

This paper shows that early and definitive career decisions to enter surgery were made by the majority of doctors who went on to work as surgeons. There are much higher rates of concordance, for surgery, between eventual destination and earlier choice than those found in other clinical specialties [8-13]. Looking backward from eventual career destinations, 90% of practising surgeons had nominated surgery as their preferred choice of career in the first year after qualification (86.2% as an untied first choice and 3.8% as a tied first choice). By contrast, only two-thirds of doctors working in the hospital medical specialties had nominated the medical specialties as their aim in the first post-qualification year; only half of general practitioners had nominated general practice as their aim in year one; and fewer than half of practising anaesthetists, radiologists, pathologists, clinical oncologists, and public health physicians had specified their eventual specialty as their choice of career when they were in their first year after qualification [13].

These findings have relevance to the structure of early surgical training, and the timing and process of selection into surgical specialties. The structure of medical training is changing worldwide with ramifications for newly qualified doctors seeking a career in surgery. In the UK, there are currently two years of general postgraduate training and work (the Foundation Programme years) before doctors can enter specialist training. There is debate over whether the two years should revert to one (the previous structure immediately after qualification), remain as two years, or indeed increase to three. A key issue for each broad specialty group, like surgery, is the timing of when doctors make the definitive decision to enter the specialty. Given that the great majority of doctors who become surgeons had decided to be surgeons in their first year after qualification, there is a case for early selection into surgery. However, there is also debate about whether selection at this stage should be directly into a surgical specialty or into a more broad-based early years programme. There are concerns that a definitive selection process at a very early stage may not be sensitive enough to accurately determine those who will go on to thrive in a surgical career. Many believe, therefore, that delaying definitive selection until after a period of core training will allow greater flexibility for trainees, as well giving an opportunity to the relatively small number of doctors who make later decisions to enter surgical training.

### Relevance to aspiring surgeons who do not progress in surgery

At years 3 and year 5 after qualification, the numbers declaring surgery as a career choice decrease substantially. It is not clear how much of the change is a consequence of active decisions on the part of the individual to change career; and how much is a consequence of being unable to enter a surgical training programme, or continue in it, despite wanting to do so (e.g. because of limited availability of training places). Lifestyle factors, such as work/life balance or intensity of out-of-hours work, have previously been shown to be significant reasons given by individuals for leaving surgery [14,15]. The early years following graduation are an important time of increasing maturity and changes of personal circumstances such as marriage and starting a family. These changes may result in an alteration in the perception of what constitutes an acceptable work/life balance.

Other individuals may be forced to consider a change of career. 'Bottlenecks' have occurred at different points in the training pathway over the last 35 years: particularly apparent was the limitation in training places at the senior house officer to registrar transition through the 1990s, corresponding to around year 3 after graduation.

It is interesting that the pattern of a decreasing proportion declaring surgery as a career choice, from year one to three to five, has not changed over time (the proportional decline is similar in the early cohorts and the more recent cohorts). Whatever the underlying reasons for this phenomenon, it has been a feature of career choice for surgery across the decades. An imperative for policymakers in surgery is to ensure that some of the brightest and most capable individuals are not being lost from the surgical specialties, for reasons that may be remediable, in these early years after graduation.

The structure of surgical training has undergone change in recent years with particular emphasis on shortening training [1-4]. In particular, *Modernising Medical Careers (MMC) *in the UK and *Surgical Education and Training (SET) *in Australasia both advocated a definitive selection into a final career specialty two years after graduation. Since its launch in 2007, the UK programme has seen a re-introduction of a second selection point at year 4/5 after graduation in most surgical specialties, following an independent inquiry investigating concerns about selection methods and the lack of flexibility in training [1]. Our data highlight some of these concerns: 40% of graduates declaring a surgical career choice did not end up working in surgery. This emphasises the importance of a broad-based early training, the value of transferable skills and the need for straightforward mechanisms of transfer between specialties. It may be that with improved selection methods the individuals most likely to become successful practising surgeons can be identified with greater accuracy. It is important that work continues on developing and validating these assessment methods.

Whilst about 20% of graduates declare a preference for surgery within the first year after graduation, in England in 2003 only about 10% of doctors in career posts (i.e. in posts as consultants or GP principals) are surgeons [7]. It is imperative that, as well as fostering the enthusiasm of those selecting a surgical career, opportunities to transfer from surgery to other specialties are provided for the considerable number of aspiring surgeons who will have to do so. It is also important that people engaged in providing advice about careers, including surgeons themselves, give soundly based information on the realistic prospects of succeeding in a career in surgery. Advice should include up-to-date information on the likely scale of availability of training and career posts.

### Trends in choice for surgery

In the 1990s and early 2000s, surgery was the preferred specialty choice of a higher percentage of medical school graduates than it had been in the previous two decades. This was in part a reflection of the decline in popularity of general practice as a career choice, but there was also an increase in the percentage of new graduates who chose surgery relative to preferences for other hospital specialties (Figure 1). The reason for this increase in popularity in the UK, made by the 1990s and subsequently sustained, is not clear. It contrasts with the decline in numbers seeking to enter general surgery residency programmes in the US over a similar period (although, in the US, numbers have begun to recover in recent years) [16].

### Choices for surgery made by men and women

As expected, significant gender differences were observed in our cohorts. The gap between the percentage of men and of women who chose surgery has narrowed over the years but is still substantial. We have reported elsewhere that women doctors who have always worked full time, as well as women who have worked part time, are under-represented in surgery [17]. Thus, under-representation is associated with female sex as well as with part time working. Given the importance of making an early and decisive choice for surgery, it is also worth noting that women who chose surgery were slightly less certain about their career choice than men, at all three time points of the surveys - one, three and five years after qualification. Furthermore, a higher percentage of women than men who eventually had careers in surgery had changed their minds from other, originally non-surgical intentions (Table 5). Reduced flexibility in moving into surgery after other early career choices would probably disadvantage women more than men.

Our results reflect the findings of other researchers, who have described sex differences in choices and career progression in hospital practice [18] and specifically in surgery [19]. It is important to ensure that numerical under-representation of women is simply a reflection of what women doctors want in terms of career choice; and that there are not remediable aspects of the specialty that deter women who might otherwise be attracted to it.

### Factors influencing choice for surgery

Doctors choosing surgery cited 'a particular teacher/department' as having a great deal of influence on specialty choice more commonly than did those opting for other hospital careers or general practice. This suggests that surgery in particular has the capacity to inspire in medical school. Undergraduate surgical teaching can often focus on the acute presentation of common surgical complaints. The rapid diagnosis-intervention-recovery pathways can be exciting; and they will appeal to students as clinical problems, and solutions, that are straightforward to conceptualise in comparison with the management of complex chronic disease. The patient's illness and recovery is often completed within the duration of the student placement. All of these factors contribute to a satisfying educational encounter for the student and, for some, will establish a particular attraction to a surgical career.

### Variation between medical schools in choice for surgery

The variation in the proportion of graduates from different medical schools who specified surgery as a career choice is interesting. A number of factors may contribute to this. The first is possible effects of differences in medical school admission policy. Many variables predict whether a candidate receives an offer from a given medical school and significant differences in the treatment of these factors by medical schools have previously been described [20]. Medical schools have been encouraged to articulate their specific objectives in a mission statement [21] and these differ between schools [22], possibly accounting for variation in the types of individuals selected to study medicine in different schools. Personality-type has been linked to eventual career choice and surgeons have been shown to have distinct temperament and personality traits [23], although this is not consistent across studies [24]. If personality is predictive of eventual specialty choice then it could be hypothesised that the admission policy of certain universities favours those with a predilection for a career in surgery. Another explanation may be the exposure of medical students to surgery in the undergraduate curriculum. The emphasis of teaching in many medical schools has moved towards primary care, with less emphasis on the hospital specialties including surgery [25], and this no doubt has occurred more at some schools than others. Early undergraduate curricula also vary and the importance placed by medical schools on different subjects varies. For instance, traditionally anatomy was regarded as an undergraduate course closely associated with the surgical specialties. A worldwide decline in the anatomy component of undergraduate courses in many medical schools has been described, possibly resulting in greater variation between schools in teaching anatomy and in fostering interests in the surgical specialties [26]. The analysis of factors influencing choice for surgery (Table 3) highlights the importance that medical students may place on the overall experience of the subject as an undergraduate and on the identification of inspirational role models. Experience of surgery, and of role models, may vary between medical schools.

## Conclusions

Surgery is a popular choice of career for medical graduates, though still much more so for men than for women. Numbers of doctors who, early in their career, would like to enter surgery exceed numbers of training posts in surgery in the UK. The great majority of doctors who progressed in a surgical career had made an early and definitive decision to do so. Of those eventually practising in surgery, 90% had expressed a first preference for it in year one and 95% had done so by year three. Unless doctors are committed to surgery very early in their careers, they are unlikely to be successful in becoming surgeons in the longer-term. Planning of postgraduate medical education, and of the timing of specialisation in surgery, should take account of the fact that most surgeons decide early on that surgery is their chosen career. There seems little point in delaying entry to surgical training - with broad-based early training - for those who have made a confident early choice for a surgical career.

## Competing interests

None. The UK Medical Careers Research Group has no financial relationships with commercial entities that might have an interest in the submitted work; has no spouses, partners, or children with relationships with commercial entities that might have an interest in the submitted work; and has no non-financial interests that may be relevant to the submitted work.

## Authors' contributions

MJG and TWL designed the study. LL and TWL analysed the data. EWH, JMJR and RWP contributed to decisions about the content of the paper and to the interpretation of the data. MJG and LL wrote the first draft. All authors contributed to further drafts and all authors read and approved the final manuscript. MJG, LL and TWL had full access to all of the data in the study, take responsibility for the integrity of the data and accuracy of the data analysis, and are guarantors.

## Pre-publication history

The pre-publication history for this paper can be accessed here:

http://www.biomedcentral.com/1471-2482/10/32/prepub

## Supplementary Material

Additional file 1 **Appendix to Table 3**. Uploaded as "SurgeryBMCAppendix to table 3.pdf". Click here for file
