# Supplementary material for: Early career choices and successful career progression in surgery in the UK: prospective cohort studies
Source: BMC Surg. 2010 Nov 2;10:32. doi: 10.1186/1471-2482-10-32 (PMC2987756; doi:10.1186/1471-2482-10-32)
Supplement: Additional file 1 — Appendix to Table 3. Uploaded as "SurgeryBMCAppendix to table 3.pdf". [file 1471-2482-10-32-S1.PDF]

Appendix to Table 3: Numbers of male and female doctors who specified each factor as influencing their choice of long-term career a great deal: one and three years (1993- 2002) and five years (1993-2000) after graduating.

	Numbers								
	Year One (1993-2002)			Year Three (1993, 1996, 2002)			Year Five (1993-2000)		
Factor	Surgery	Other Hospital	GP	Surgery	Other Hospital	GP	Surgery	Other Hospital	GP
Men									
Domestic circumstances	163	319	363	96	247	302	198	566	674
Hours/ working conditions	390	996	736	225	643	529	290	944	912
Future financial prospects	454	404	197	207	202	194	225	275	265
Career & promotion prospects	547	835	225	235	491	206	311	706	290
Self-appraisal of own skills/ aptitudes	928	1520	447	415	915	335	583	1334	546
Advice from others	315	479	161	158	273	102	186	345	129
Experience of chosen subject as a student	892	1339	368	222	412	144	256	495	181
A particular teacher/ department#	641	832	128	171	324	53	183	288	25
Inclinations before medical school	374	343	150	167	171	105	169	214	121
Experience of jobs so far	1080	1463	452	578	1144	339	761	1587	552
Enthusiasm/ Commitment	1383	1825	559	583	1068	378	849	1721	638
Women									
Domestic circumstances	83	658	954	40	464	771	96	1472	1945
Hours/ working conditions	223	1534	1859	101	961	1286	139	1472	1945
Future financial prospects	94	316	355	25	155	308	18	151	315
Career & promotion prospects	202	943	355	65	488	296	67	598	374
Self-appraisal of own skills/ aptitudes	449	2022	1178	145	1178	767	216	1672	1172
Advice from others	170	706	408	60	386	264	64	398	229
Experience of chosen subject as a student	461	1972	928	125	655	315	123	660	392
A particular teacher/ department#	377	1283	341	102	465	106	77	368	64
Inclinations before medical school	130	581	367	50	284	222	41	264	301
Experience of jobs so far	566	2153	1249	253	1625	859	271	1999	1197
Enthusiasm/ Commitment	682	2765	1511	262	1550	867	321	2222	1375
